# Tratamento Ambulatorial de Levosimedana por 6 Horas como Ponte para Transplante Cardíaco

**DOI:** 10.36660/abc.20220205

**Published:** 2023-02-13

**Authors:** António Valentim Gonçalves, João Pedro Reis, Ana Teresa Timóteo, Rui Soares, Tiago Pereira-da-Silva, Valdemar Gomes, Rita Ilhão Moreira, Delmira Pombo, Tiago Carvalho, Catarina Correia, Claudia Santos, Rui Cruz Ferreira

**Affiliations:** 1 Departamento de Cardiologia Hospital de Santa Marta Centro Hospitalar Universitário de Lisboa Central Lisboa Portugal Departamento de Cardiologia – Hospital de Santa Marta – Centro Hospitalar Universitário de Lisboa Central, Lisboa – Portugal

**Keywords:** Levosimedana, Insuficiência Cardíaca, Tratamento em Hospital de Dia, Ponte para Transplante

## Resumo

Registro observacional que inclui 8 pacientes consecutivos que aguardavam um transplante cardíaco e necessitaram de terapia inotrópica permanente ou regular (INTERMACS classe 3 ou 4) entre 2018 e 2021. Levosimedana foi administrado em infusão intravenosa de 6 horas (0,2μg/kg/min) a cada 2 semanas em ambiente ambulatorial. O tratamento foi continuado até que um doador estivesse disponível, ou a melhora clínica o tornasse desnecessário.

O seguimento médio foi de 4,1±3,5 meses, e nenhum paciente necessitou de outra internação enquanto aguardava doador. Os tratamentos foram bem tolerados, sem eventos adversos. Um paciente melhorou, enquanto os outros 7 pacientes foram transplantados sem qualquer sinal de vasoplegia após a cirurgia cardíaca. Todos os pacientes estavam em casa e vivos 30 dias após o transplante cardíaco.

Até onde sabemos, este é o primeiro relato da possibilidade de fazer uma ponte em pacientes em INTERMACS classe III ou IV com tratamento de Levosimedana por 6 horas sem a necessidade de hospitalizações recorrentes.

O transplante cardíaco é o tratamento de escolha para pacientes selecionados com insuficiência cardíaca (IC) avançada, melhorando a qualidade de vida e conferindo benefício de sobrevida em comparação ao manejo convencional.^1^ No entanto, o transplante cardíaco depende da presença de um doador, necessitando de estratégias de ponte. A infusão de inotrópicos foi sugerida como um desses tratamentos, mas uma metanálise de estudos randomizados encontrou aumento da mortalidade com suporte inotrópico de longo prazo,^2^ e as diretrizes de IC não endossam essa estratégia.

O inodilatador Levosimedana surgiu como opção de tratamento: sua ação inotrópica pode se prolongar por até 2 semanas, sendo menos pró-arrítmica que os inotrópicos tradicionais.^3^ Era tradicionalmente administrado como uma infusão de 24 horas. No entanto, uma infusão de 24 horas requer pelo menos uma internação de dois dias. Recentemente, dois ensaios clínicos duplo-cegos randomizados controlados por placebo avaliaram ciclos de 6 horas de Levosimedana em pacientes ambulatoriais com IC avançada e demonstraram que reduz os níveis de NT-proBNP e internações por IC aguda descompensada.^4 , 5^

Até onde sabemos, este é o primeiro relato de uma infusão ambulatorial de Levosimedana de 6 horas sendo usada como ponte para transplante cardíaco em pacientes classificados como INTERMACS classe 3 ou 4.

Este é um registro observacional de centro único que inclui 8 pacientes consecutivos que aguardavam um transplante cardíaco e necessitaram de terapia inotrópica permanente ou regular (INTERMACS classe 3 ou 4) entre 2018 e 2021. A investigação está em conformidade com os princípios descritos na Declaração de Helsinque. Todos os pacientes forneceram consentimento informado por escrito.

Levosimedana foi administrada em infusão intravenosa de 6 horas (0,2μg/kg/min, sem bolus) a cada duas semanas em ambiente ambulatorial com monitoramento não invasivo dos sinais vitais. A terapia para IC foi mantida durante o dia do procedimento. No primeiro ciclo de tratamento, foi iniciada uma dose de 0,05 μg/kg/min, titulada para 0,1 μg/kg/min se bem tolerado nas primeiras duas horas, e para 0,2 μg/kg/min após mais duas horas de tratamento.

O tratamento a cada duas semanas foi continuado até que um doador estivesse disponível ou uma melhora clínica significativa tornasse o tratamento desnecessário.

O seguimento médio foi de 4,1 ± 3,5 meses, com três pacientes com cardiomiopatia isquêmica, duas cardiomiopatias induzidas por quimioterapia, uma cardiomiopatia arritmogênica e duas cardiomiopatias dilatadas, uma associada a doença valvular prévia e uma idiopática. As características populacionais basais detalhadas são descritas na Tabela 1 , revelando uma população com IC avançada (mediana de NTproBNP 7595pg/ml, índice cardíaco médio 1,8 L/min/m^2^ e pico médio de consumo de oxigênio previsto de 41%). Devido à sua dependência de medicação inotrópica permanente ou regular para estabilidade clínica, todos os pacientes foram classificados como INTERMACS classe 3 ou 4 antes de iniciar tratamentos de 6 horas com Levosimedana.

**Tabela 1:** – Características basais da população do estudo (n=8)

Idade (anos)	55,6 ± 16,2
Etiologia isquêmica	3 (37,5%)
Sexo masculino	3 (37,5%)
Pressão arterial sistólica (mmHg)	102,0 ± 18,4
Frequência cardíaca (bpm)	69,5 ± 9,4
Furosemida dose diária (mg)	100,0 ± 51,3
Taxa de filtração glomerular (ml/min)	55,9 ± 25,6
NTproBNP (pg/ml)	7595 (3956, 12038)
Diâmetro diastólico final do ventrículo esquerdo (mm)	67,3 ± 14,7
Diâmetro sistólico final do ventrículo esquerdo (mm)	55,8 ± 15,9
Fração de ejeção do ventrículo esquerdo (%)	29,9 ± 6,9
Strain longitudinal global (%)	6,9 ± 4,1
Débito cardíaco (l/min)	3,3 ± 0,5
Índice cardíaco (l/min/m_2_)	1,8 ± 0,3
Pressão capilar pulmonar em cunha (mmHg)	23,3 ± 7,4
Pressão atrial direita (mmHg)	12,1 ± 7,1
Pressão média da artéria pulmonar (mmHg)	32,3 ± 9,6
Resistência vascular pulmonar (WU)	2,7 ± 1,5
Índice de pulsatilidade da artéria pulmonar	3,3 ± 2,4
Trabalho cardíaco	0,6 ± 0,2
Consumo máximo de oxigênio (ml/kg/min)	12,0 ± 2,0
Consumo máximo de oxigênio previsto (%)	40,8 ± 6,0
Inclinação VE/VCO_2_	46,6 ± 5,4
Pico de fluxo expiratório	1,0 ± 0,1

As infusões foram hemodinamicamente bem toleradas (média de 10 tratamentos por paciente). Não houve efeitos adversos graves, como arritmias sustentadas, infecções associadas ao acesso venoso periférico ou hipotensão sintomática, que exigissem redução da dose padrão de 0,2 g/kg/min.

Nenhum paciente necessitou de outra internação enquanto aguardava um transplante cardíaco. Um paciente melhorou durante o programa Levosimedana e foi removido da lista de transplante cardíaco. Os outros 7 pacientes foram transplantados com sucesso.

Quando NTproBNP, troponina e taxa de filtração glomerular foram comparados entre o primeiro ciclo de tratamento ambulatorial com Levosimedana e o último ciclo antes de um coração ficar disponível, houve uma redução numérica em NTproBNP (7595 (3956, 12038) pg/ml vs. 5415 (2713, 10263) pg/ml) e troponina (32,2 ± 26,1 pg/ml vs. 22,3 ± 10,3 pg/ml), enquanto a taxa de filtração glomerular aumentou numericamente (55,9 ± 25,6 ml/min vs. 61,2 ± 23,3 ml/min).

O transplante cardíaco foi realizado em média 6,3 ± 4,5 dias após o último tratamento ambulatorial com Levosimedana. Um paciente tinha um coração disponível no dia do tratamento. Esses pacientes não apresentaram sinais de vasoplegia após a cirurgia cardíaca. Ao utilizar nosso protocolo inotrópico padrão com Isoprenalina conforme necessário para frequência cardíaca acima de 90bpm nas primeiras 72 horas e Dobutamina (2,5 g/kg/min) ou Milrinona (0,2 g/kg/min) de acordo com a preferência do cirurgião cardíaco em carga do procedimento, a pressão arterial média 24 horas após o procedimento foi de 82,1 ± 8,2mmHg. Nenhum paciente teve suporte inotrópico 96 horas após o procedimento. Todos os pacientes estavam em casa e vivos 30 dias após o transplante cardíaco.

Esses achados preliminares destacam a possibilidade de conectar pacientes em INTERMACS classe III ou IV com tratamento de 6 horas com Levosimedana sem a necessidade de hospitalizações recorrentes.
