# Supplementary material for: Tratamento Ambulatorial de Levosimedana por 6 Horas como Ponte para Transplante Cardíaco
Source: Arq Bras Cardiol. 2023 Feb 13;120(2):e20220205. [Article in Portuguese] doi: 10.36660/abc.20220205 (PMC9972780; doi:10.36660/abc.20220205)
Supplement: Translation

# Translation: Outpatient 6-Hour Levosimendan Treatment as a Bridge to Heart Transplant

**Authors:** António Valentim Gonçalves, João Pedro Reis, Ana Teresa Timóteo, Rui Soares, Tiago Pereira-da-Silva, Valdemar Gomes, Rita Ilhão Moreira, Delmira Pombo, Tiago Carvalho, Catarina Correia, Claudia Santos, Rui Cruz Ferreira

**Affiliations:** 1 Portugal Departamento de Cardiologia – Hospital de Santa Marta – Centro Hospitalar Universitário de Lisboa Central, Lisboa – Portugal

**Keywords:** Levosimendan, Heart Failure, Outpatient Treatment, Bridge to Transplant

Heart transplantation is the treatment of choice for selected patients with advanced heart failure (HF), improving quality of life and conferring a survival benefit compared to conventional management.^1^However, heart transplantation depends on a donor’s presence, necessitating bridge strategies. Infusion of inotropes was suggested as one of these treatments, but a meta-analysis of randomized trials found increased mortality with long-term inotropic support,^2^and HF guidelines do not now endorse this strategy.

The inodilator Levosimendan has arisen as a treatment option: its inotropic action can continue for up to 2 weeks while being less pro-arrhythmic than traditional inotropes.^3^It was traditionally administered as a 24-hour infusion. Nonetheless, a 24-hour infusion necessitates at least a two-day stay in the hospital. Recently, two randomized placebo-controlled, double-blind clinical trials evaluated 6-hour cycles of Levosimendan therapy in ambulatory patients with advanced HF and demonstrated that it reduces NT-proBNP levels and admissions for acute decompensated HF.^4 , 5^

To the best of our knowledge, this is the first report of an outpatient 6-hour Levosimendan infusion being used to bridge to heart transplant in patients classified as INTERMACS class 3 or 4.

This single-center observational registry includes 8 consecutive patients waiting for a heart transplant and required permanent or regular inotropic therapy (INTERMACS class 3 or 4) between 2018 and 2021. The investigation conforms to the principles outlined in the Declaration of Helsinki. All patients provided written informed consent.

Levosimendan was given as a 6-hour intravenous infusion (0.2 μg/kg/min, without bolus) every two weeks in an ambulatory setting with non-invasive monitoring of vital signs. HF therapy was maintained during the day of the procedure. In the first treatment cycle, a dose of 0.05 μg/kg/min was started, titrated to 0.1 μg/kg/min if well tolerated in the first two hours, and to 0.2 μg/kg/min after another two hours of treatment.

Treatment every two weeks was continued until a donor was available or a significant clinical improvement made the treatment unnecessary.

The mean follow-up was 4.1 ± 3.5 months, with three patients with ischemic cardiomyopathy, two chemotherapy-induced cardiomyopathies, one arrhythmogenic cardiomyopathy, and two dilated cardiomyopathy, one associated with previous valvular disease and one idiopathic. Detailed baseline population characteristics are described in Table 1 , revealing an advanced HF population (median NTproBNP 7595pg/ml, mean cardiac index 1.8 L/min/m^2^and mean peak predicted oxygen consumption of 41%). Due to their reliance on permanent or regular inotropic medication for clinical stability, all patients were classified as INTERMACS class 3 or 4 before starting 6-hour Levosimendan treatments.

**Table 1:** – Baseline characteristics of the study population (n=8)

Age (years)	55.6 ± 16.2
Ischemic etiology	3 (37.5%)
Male gender	3 (37.5%)
Systolic blood pressure (mmHg)	102.0 ± 18.4
Heart rate (bpm)	69.5 ± 9.4
Furosemide daily dose (mg)	100.0 ± 51.3
Glomerular Filtration Rate (ml/min)	55.9 ± 25.6
NTproBNP (pg/ml)	7595 (3956, 12038)
Left ventricle end-diastolic diameter (mm)	67.3 ± 14.7
Left ventricle end-systolic diameter (mm)	55.8 ± 15.9
Left ventricular ejection fraction (%)	29.9 ± 6.9
Global longitudinal strain (%)	6.9 ± 4.1
Cardiac output (l/min)	3.3 ± 0.5
Cardiac index (l/min/m^2^)	1.8 ± 0.3
Pulmonary capillary wedge pressure (mmHg)	23.3 ± 7.4
Right atrial pressure (mmHg)	12.1 ± 7.1
Mean pulmonary artery pressure (mmHg)	32.3 ± 9.6
Pulmonary vascular resistance (WU)	2.7 ± 1.5
Pulmonary artery pulsatility index	3.3 ± 2.4
Cardiac power output	0.6 ± 0.2
Peak oxygen consumption (ml/kg/min)	12.0 ± 2.0
Peak predicted oxygen consumption (%)	40.8 ± 6.0
VE/VCO_2_slope	46.6 ± 5.4
Peak ratio exchange ratio	1.0 ± 0.1

Infusions were hemodynamically well tolerated (mean 10 treatments for patient). There were no severe adverse effects, such as sustained arrhythmias, infections linked with peripheral venous access, or symptomatic hypotension, that required a reduction in the 0.2 g/kg/min standard dose.

No patient needed other hospitalization while waiting for a heart transplant. One patient improved during the Levosimendan program and was removed from the heart transplant list. The other 7 patients were successfully transplanted.

When NTproBNP, troponin, and glomerular filtration rate were compared between the first cycle of outpatient Levosimendan treatment and the last cycle before a heart became available, there was a numerical reduction in NTproBNP (7595 (3956, 12038) pg/ml vs. 5415 (2713, 10263) pg/ml) and troponin (32.2 ± 26.1 pg/ml vs. 22.3 ± 10.3 pg/ml), while the glomerular filtration rate numerically increased (55.9 ± 25.6 ml/min vs. 61.2 ± 23.3 ml/min).

Heart transplant was performed at a mean of 6.3 ± 4.5 days after the last outpatient Levosimendan treatment. One patient had a heart available on the day of the treatment. These patients had no signs of vasoplegia after the cardiac surgery. While using our standard inotropic protocol with Isoprenaline as needed for a heart rate above 90bpm in the first 72 hours and Dobutamine (2.5 g/kg/min) or Milrinone (0.2 g/kg/min) according to the preference of the cardiac surgeon in charge of the procedure, mean blood pressure 24 hours after the procedure was 82.1 ± 8.2mmHg. No patient had inotropic support 96 hours after the procedure. All patients were at home and alive 30 days after the heart transplant.

These preliminary findings highlight the possibility of bridging patients in INTERMACS class III or IV with 6-hour Levosimendan treatment without requiring recurrent hospitalizations.
